# Intraoperative acute correction versus postoperative gradual correction for tibial shaft fractures with multiplanar posttraumatic deformities using the hexapod external fixator

**DOI:** 10.1186/s12891-021-04505-0

**Published:** 2021-09-18

**Authors:** Yanshi Liu, Feiyu Cai, Kai Liu, Xingpeng Zhang, Hong Li, Xuefei Fu, Tao Zhang, Aihemaitijiang Yusufu

**Affiliations:** 1 Department of Trauma and Microreconstructive Surgery, the First Affiliated Hospital of Xinjiang Medical University, Urumqi, Xinjiang China; 2 Department of Orthopedics, Shanghai Pudong New Area People’s Hospital, Shanghai, China; 3 Department of Orthopedics, Zigong Fourth People’s , Hospital, Zigong, Sichuan China; 4 Department of Orthopedics, Anhui No. 2 Provincial People’s Hospital, Hefei, Anhui China; 5 Department of Orthopedics and Trauma, Tianjin Hospital, Tianjin, China

**Keywords:** Acute correction, Deformity correction, Gradual correction, Tibial shaft fractures, Hexapod external fixator

## Abstract

**Background:**

The purpose of this study was to determine the differences in clinical outcomes, if any, between intraoperative acute correction and postoperative gradual correction for tibial shaft fractures with multiplanar posttraumatic deformities using the hexapod external fixator.

**Methods:**

We retrospectively analyzed 58 consecutive patients with tibial shaft fractures treated by the hexapod external fixator at our institution from January 2015 to April 2019. Twenty-three patients (Group I) underwent intraoperative acute correction, from January 2015 to October 2016. Starting in November 2016, the other 35 patients (Group II) all underwent postoperative gradual correction. The demographic data, operation duration, original residual deformities before correction, residual deformities after correction, and external fixation time were collected and analyzed. The clinical outcomes were evaluated by the Johner-Wruhs criteria at the last clinical visit.

**Results:**

All patients achieved complete bone union with a mean time of 28.7 ± 4.6 weeks (range 21 to 37 weeks) in Group I and 27.9 ± 4.8 weeks (range 19 to 38 weeks) in Group II (*P* > 0.05). The operation duration in Group I (88.9 ± 7.7 min) was longer than that in Group II (61.9 ± 8.4 min), and there was a statistically significant difference (*P* < 0.05). There were no statistically significant differences between the two groups in original residual deformities before correction and residual deformities after correction (*P* > 0.05). The rate of postoperative complication was similar between the two groups. There was no statistical significance in demographic data and clinical outcomes between the two groups (*P* > 0.05).

**Conclusions:**

There is no difference in clinical outcomes between intraoperative acute correction and postoperative gradual correction for tibial shaft fractures with multiplanar posttraumatic deformities using the hexapod external fixator. Postoperative gradual correction may shorten the duration in the operation room and decrease the potential intraoperative risk.

## Background

The hexapod external fixator (HEF), such as the Taylor spatial frame (TSF), consisting of 2 full or partial rings connected by 6 telescopic struts at special universal joints, is a practical tool for limb deformity correction [1-3]. The HEF provides all the advantages of multiplanar fixation and is equipped with the versatility of spatial deformities correction without altering the frame. As more general orthopedic surgeons are gaining expertise in this versatile device, the HEF has become an attractive option for trauma-control and definitive management, especially in fractures with poor surrounding soft tissues [4-9].

For the management of high-energy lower limb trauma, the external fixator has the advantages of stable fixation, minimal soft tissue disruption, and early weight-bearing [10]. The HEF is commonly acute used for tibial fractures, stabilizing closed or open fractures [5-7, 11] and allowing anatomic realignment. Optimal limb alignment is the goal of reconstructive surgery, and it is possible to “run” the residual deformity program to achieve “finetuning” of bone alignment before bone union using the HEF. Although satisfactory clinical outcomes have been manifested by both intraoperative acute correction and postoperative gradual correction respectively, we could not find any study comparing clinical results of both methods, and the superiority remains uncertain. The purpose of this study was to determine the differences in clinical outcomes, if any, between intraoperative acute correction and postoperative gradual correction for tibial shaft fractures with multiplanar posttraumatic deformities using the hexapod external fixator.

## Methods

This retrospective study included 58 patients with tibial shaft fractures, who were admitted to our department and consented to hexapod external fixator (Tianjin Xinzhong Medical Instrument Co., Ltd., Tianjin, China) treatment from January 2015 to April 2019. Inclusion criteria were open fractures and closed fractures with poor surrounding soft tissues. Patients with pathological fractures, age > 65, poor compliance, and patients treated initially with the HEF but converted to internal fixation were excluded. Informed consent was obtained from all patients for their data to be documented and published in our study. The Ethical Committee of our institution approved this study.

Twenty-three patients (Group I) underwent intraoperative acute correction, from January 2015 to October 2016. There were 20 males and 3 females with an average age of 39 years (range 19 to 64 years), including 13 left extremities and 10 right extremities. This group included 15 open fractures and 8 closed fractures. The fractures were divided by the OTA classification system. The injury mechanism was road traffic accident in 17 patients, fall from height in 4 patients, and crushing injury in 2 patients. The mean time elapsed since the injury to the HEF installed was 3.9 days (range 1 to 8 days). The physiological instability and associated soft-tissue injury contributed to the delayed installation of the HEF.

Starting in November 2016, the other 35 patients (Group II) all underwent postoperative gradual correction. This group included 27 males and 8 females with a mean age of 41 years (range 26 to 61 years), containing 21 left extremities and 14 right extremities. There were 24 open fractures and 11 closed fractures. The injury mechanism was road traffic accident in 25 patients, fall from height in 7 patients, and crushing injury in 3 patients. The mean time elapsed since the injury to the HEF installed was 3.2 days (range 1 to 9 days).

The same medical team performed all the surgical procedures. The demographic data, operation duration, original residual deformities before correction, residual deformities after correction, and external fixation time were collected and analyzed. The intraoperative and perioperative difficulties were subclassified according to Paley [12]. The clinical outcomes were evaluated by the Johner-Wruhs criteria [13] at the last clinical visit.

### The technique of deformity correction

For intraoperative acute correction, the HEF was installed using a method similar to Gantsoudes et al. [14]. The reference ring must be mounted vertically to the long axis of the proximal bony fragment. The deformity parameters were measured using intraoperative fluoroscopy. For mounting parameters, one nut was placed firstly at the midpoints of the anteroposterior (AP) view on the reference ring. Subsequently, the anterior marker (a rod with a cube) was placed at the center hole of the master tab (anterior tab) on the reference ring. By means of slowly rotating the limb, projections of these two markers were overlapped under static or live fluoroscopy, and the distance of the rod (representing the center of the ring) to the center of the tibia was measured with a sterile ruler directly off of the cube. The AP view frame offset and lateral view frame offset were measured by the method mentioned above. The axial view frame offset was calculated by the distance from the reference ring to the origin point. All the parameters were entered in the computer program, and the residual deformities were corrected acutely depending on the electronic prescription.

As for postoperative gradual correction, the “ring-first” technique was used in all the patients. The reference ring was perpendicular to the long axis of the corresponding bony segment in an orthogonal manner. Standard postoperative orthogonal AP and lateral radiographs were used to evaluate the residual deformities and calculate the mounting parameters. The AP view frame offset was measured using the distance of the line perpendicular to the anatomical axis of the reference bony fragment from the center of the reference ring to the origin point. The lateral view frame offset was calculated in the same way. The axial view frame offset was calculated using the distance of the line parallel to the anatomical axis of the reference bony fragment from the center of the reference ring to the origin point. All the residual deformities were corrected by gradual strut adjustment postoperatively within seven days, according to the electronic prescription.

### Postoperative management

The translation and angulation in the coronal and sagittal plane, according to the standard AP and lateral X-rays after correction, were used to evaluate the effectiveness. Isometric muscle and joint range of motion exercises were recommended for all patients on the second day after the operation. The foot was kept in a neutral position to prevent ankle equines contractures using a rigid shoe with an elastic band. Daily pin site care with alcoholic chlorhexidine was performed to avoid pin tract infection.

The patients were followed up and taken a radiograph monthly until the bone union was achieved. The HEF was removed when sufficient union (corticalization in 3 of 4 cortices) was shown in radiographs. The functional brace was used to prevent refracture for four weeks after frame removal in all patients.

### Statistical analysis

Statistical analysis was performed with the SPSS 22.0 (IBM Corp, USA). Continuous variables were analyzed by Independent-samples T-tests and expressed as the mean, standard deviation, and range of the observations. And the count variables were analyzed by the Chi-square or Fisher’s test and expressed as a number. A statistically significant difference was set at P < 0.05.

## Results

**Fig. 1:**
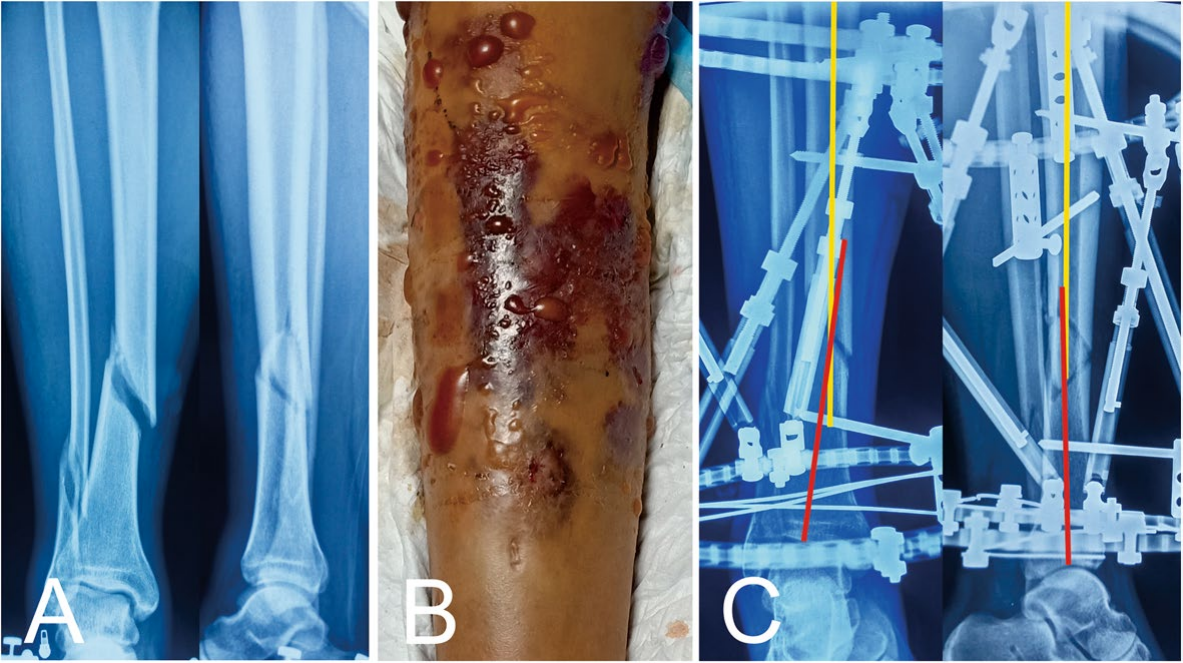
Images of a 43-year-old man with posttraumatic multidimensional deformities in tibia and fibula treated by the HEF. **a** Posttraumatic AP and lateral views of radiographs. **b** Patient with preoperative hemorrhagic fracture blisters seen and poor surrounding soft tissues. **c** Intraoperative AP and lateral radiographs, showing the residual deformities that needed to be corrected

**Fig. 2:**
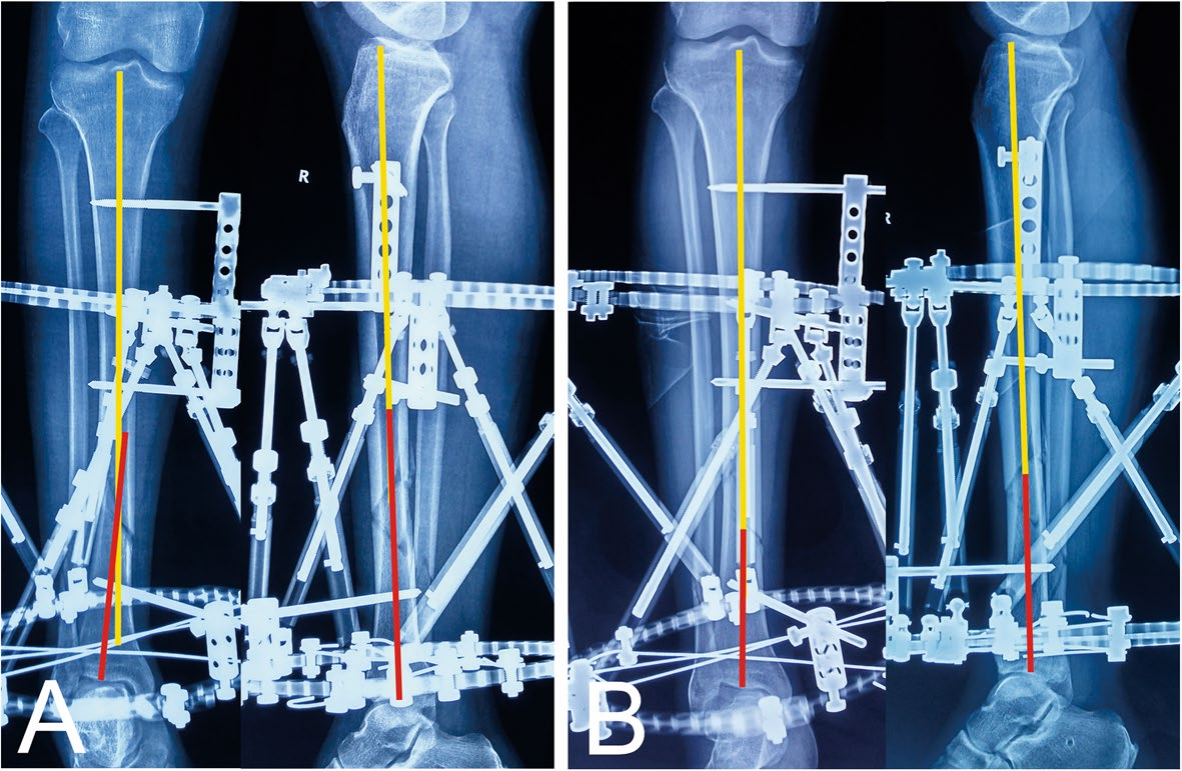
Images of the same patient shown in Fig. 1. **a** Radiographs immediately after operation. **b** Radiographs after final correction

**Fig. 3:**
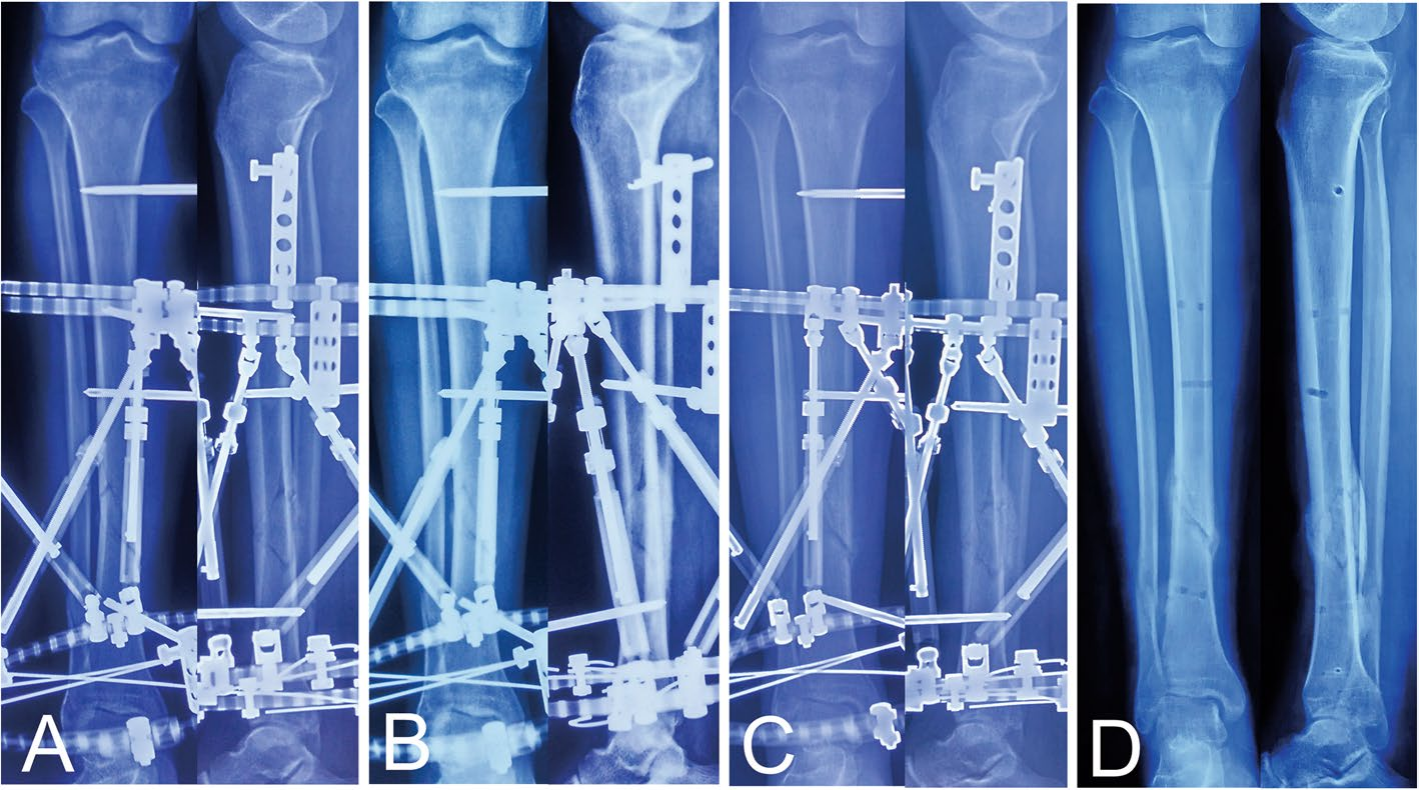
Follow-up radiographs of the same patient after final correction. **a** Radiographs one month later. **b** Radiographs three months later. **c** Radiographs five months later. **d** Radiographs three months later after the frame removal

The demographic data of the two groups are shown in Table 1, and there are no statistical significances (*P* > 0.05). The clinical outcomes are shown in Table 2 and Table 3. The typical case of postoperative gradual correction is shown in Figs. 1, 2 and 3.

**Table1:** Demographic data of the two groups

Variable	Group I	Group II	Statistical value	*P* value
Patients	23	35	-	-
Gender				
Male	20	27	-	0.499
Female	3	8		
Age (year)	39.0 ± 11.6	41.9 ± 10.4	-1.001	0.321
Injury mechanism				
Road traffic accident	17	25	0.197	1.000
Fall from height	4	7		
Crushing injury	2	3		
Open/closed fracture				
Open	15	24	0.071	0.790
Closed	8	11		
Injured bone				
Left tibia	13	21	0.069	0.792
Right tibia	10	14		
OTA classification of fractures				
A	5	9	0.626	0.785
B	16	21		
C	2	5		
The time elapsed since the injury to HEF installed (day)	3.9 ± 2.2	3.2 ± 2.2	1.220	0.228

HEF: hexapod external fixator

**Table 2:** Clinical outcomes of the two groups

Variable	Group I	Group II	Statistical value	*P* value
Operation duration (Min)	88.9 ± 7.7	61.9 ± 8.4	12.397	*P* < 0.001
Residual deformities before correction				
T1(mm)	8.9 ± 5.5	9.3 ± 5.3	-0.326	0.745
A1(°)	5.4 ± 2.6	5.1 ± 3.1	0.405	0.687
T2(mm)	9.1 ± 2.7	8.2 ± 5.1	0.759	0.451
A2(°)	3.6 ± 2.0	4.3 ± 2.1	-1.162	0.250
Residual deformities after correction				
T1(mm)	1.7 ± 1.3	1.5 ± 1.7	0.568	0.572
A1(°)	0.6 ± 0.8	0.5 ± 0.7	0.704	0.484
T2(mm)	1.2 ± 0.9	0.9 ± 1.3	1.281	0.206
A2(°)	0.5 ± 0.7	0.7 ± 1.0	-1.160	0.251
External fixation time (week)	28.7 ± 4.6	27.9 ± 4.8	0.587	0.560
Follow-up (month)	17.3 ± 3.3	16.1 ± 4.3	1.135	0.261
Johner-Wruhs criteria				
Excellent	18	28	0.343	1.000
Good	4	5		
Moderate	1	2		
Poor	0	0		

T1: Residual translation on the AP view

A1: Residual angulation on the AP view

T2: Residual translation on the lateral view

A2: Residual angulation on the lateral view

**Table 3:** Complications in the two groups

	Group I		Group II	
Complications	Number	Percentage (%)	Number	Percentage (%)
Pin tract infection	9	39.1%	12	34.3%
Delayed union	2	8.7%	2	5.7%
Joint stiffness	1	4.3%	2	5.7%
Total	12		16	
Total patients affected	8		11	
Complication rate	34.8%		31.4%

The operation duration in Group I was 88.9 ± 7.7 min, while 61.9 ± 8.4 min in Group II (*P* < 0.05). There were no statistically significant differences between the two groups in original residual deformities before correction and residual deformities after correction (*P* > 0.05). All patients achieved complete bone union with a mean time of 28.7 ± 4.6 weeks (range 21 to 37 weeks) in Group I and 27.9 ± 4.8 weeks (range 19 to 38 weeks) in Group II (*P* > 0.05).

There were no complications during the operation. After deformity correction, no compartment syndrome was observed in both groups. Twelve patients who underwent postoperative gradual correction felt pain during the procedure and managed by the oral analgesics. Postoperative pin tract infection was commonly observed in the present study. Nine patients in Group I (39.1%) and twelve patients in Group II (34.3%) suffered superficial pin tract infection, and successfully managed by daily pin site care and oral antibiotics. None suffered deep pin tract infection and developed sequestrum requiring debridement.

There were two patients in both Group I (8.7%) and Group II (5.7%) who suffered delayed union, and were successfully treated by the “accordion maneuver” technique. The frames were thereby terminated at 35 weeks and 37 weeks in Group I, 37 weeks and 38 weeks in Group II.

Ankle joint stiffness after frame removal was observed in one case (4.3%) for Group I and two cases for Group II (5.7%), and finally managed by a surgical release. No patients of the two groups developed a loss of reduction, a malunion, and refracture.

All patients were successfully followed up at least 12 months after the HEF removal, and none was lost (17.3 ± 3.3 months in Group I, 16.1 ± 4.3 months in Group II, *P* > 0.05). All the patients were able to perform daily activities without significant difficulties at the last clinical visit. According to the Johner-Wruhs criteria, there were excellent in 18 patients, good in 4 patients, and moderate in 1 patient in Group I. As for Group II, there were excellent in 28 patients, good in 5, and moderate in 2 (*P* > 0.05).

## Discussion

The hexapod external fixator is an evolution of circular fixation that relies upon the Ilizarov technique and adds the Chasles theorem of 6-axis motion [14, 15]. Combining the frame modularity and the supporting computer program makes the HEF a powerful tool for deformity correction. Initially developed for multiplanar deformities correction, the HEF subsequently expanded to manage the fractures and bone nonunion [4-6, 16-19] as more general orthopedic surgeons are becoming familiar with this device.

Although the hexapod external fixator is mainly applied for gradual deformity correction, it also can be used for acute deformity correction. However, no technique is perfect in fact, as currently most measurement techniques heavily rely on human evaluators. Optimal deformity correction is dependent on the accurate definition of the deformity and mounting parameters together with the length of the six struts. Furthermore, even subtle errors in the parameters can be showed as malcorrection, unexpected translation-angulation, or insufficient correction. In the previously published data, various methods have been proposed to improve the correction accuracy, including intraoperative fluoroscopy, postoperative radiography, and CT scans [14, 20-24].

Gantsoudes et al. [14] described an intraoperative quick and cheap technique that utilized equipment already available in virtually all settings in which a TSF would be used, declaring it is reproducible easily in the operating room and allows for accurate measurement. Kucukkaya et al. [22] acquired the mounting parameters based on the CT scans, and it was especially suitable for cases with a rotational deformity. Deakin DE et al. [23] used a frame-mounted spirit level to help the radiographer produce perfectly aligned radiographs. Ahrend et al. [21] conducted postoperative radiographs with the help of a rotation rod to decrease the variability of rotation on radiographs. Wright et al. [25] also developed a silhouette technique to obtain adequate orthogonal imaging and reduce the repeated radiograph requirement. Liu et al. [24] combined the elliptic registration technique and three-dimensional reconstruction to precisely measure the parameters.

Although all the aforementioned methods aimed to the accurate parameter measurement, they focused solely on the deformity correction itself, and none compared the clinical outcomes of intraoperative acute correction versus postoperative gradual correction.

In the present study, we retrospectively analyzed 58 patients with tibial shaft fractures treated by the hexapod external fixator. Twenty-three patients underwent intraoperative acute correction, and the other 35 patients underwent postoperative gradual correction. The results manifested that there were no statistically significant differences in demographic data and clinical outcomes between the two groups, and the rate of postoperative complication was similar. However, the operation duration in Group I (88.9 ± 7.7 min) was longer than that in Group II (61.9 ± 8.4 min). Although 12 patients felt pain during the postoperative gradual correction, the oral analgesics was sufficient and did not require additional intervention.

The ability to acquire accurate parameters will be affected by certain factors and may result in other new deformities during the correction, especially in the operating room. For the hexapod external fixator, one of the major advantages is performing further residual corrections as needed. In addition, each residual correction is more accurate than the last. Furthermore, the worth-considering problem is that the intraoperative acute deformity correction may lead to increased tension in the osteofascial compartments causing a compartment syndrome that needed additional surgical intervention and is harmful for bone regeneration. Therefore, according to our experience, both the intraoperative acute correction and postoperative gradual correction can achieve satisfactory clinical outcomes, while the postoperative gradual correction is recommended due to the shorter surgery duration with lower potential intraoperative risk.

The present study may be limited by the retrospective nature with a single-center small sample size, a conservative attitude therefore should be adopted regarding the interpretations of our results. Despite this inherent limitation, this study firstly conducts a comparison between intraoperative acute correction and postoperative gradual correction for tibial shaft fractures with multiplanar posttraumatic deformities using the hexapod external fixator, and draws a preliminary conclusion.

## Conclusion

There is no difference in clinical outcomes between intraoperative acute correction and postoperative gradual correction for tibial shaft fractures with multiplanar posttraumatic deformities using the hexapod external fixator. Postoperative gradual correction may shorten the duration in the operation room and decrease the potential intraoperative risk.

## Data Availability

The datasets analysed during the current study are available from the corresponding author on reasonable request.

## Abbreviations

HEF: Hexapod external fixator
TSF: Taylor spatial frame
AP: Anteroposterior
